# Comparison of vaccine-induced antibody neutralization against SARS-CoV-2 variants of concern following primary and booster doses of COVID-19 vaccines

**DOI:** 10.3389/fmed.2022.994160

**Published:** 2022-10-03

**Authors:** Astrid K. Hvidt, Eva A. M. Baerends, Ole S. Søgaard, Nina B. Stærke, Dorthe Raben, Joanne Reekie, Henrik Nielsen, Isik S. Johansen, Lothar Wiese, Thomas L. Benfield, Kasper K. Iversen, Ahmed B. Mustafa, Maria R. Juhl, Kristine T. Petersen, Sisse R. Ostrowski, Susan O. Lindvig, Line D. Rasmussen, Marianne H. Schleimann, Sidsel D. Andersen, Anna K. Juhl, Lisa L. Dietz, Signe R. Andreasen, Jens Lundgren, Lars Østergaard, Martin Tolstrup, the ENFORCE Study Group

**Affiliations:** ^1^Department of Infectious Diseases, Aarhus University Hospital, Aarhus, Denmark; ^2^Department of Clinical Medicine, Aarhus University, Aarhus, Denmark; ^3^Center of Excellence for Health, Immunity and Infections, Rigshospitalet, University of Copenhagen, Copenhagen, Denmark; ^4^Department of Infectious Diseases, Aalborg University Hospital, Aalborg, Denmark; ^5^Department of Clinical Medicine, Aalborg University, Aalborg, Denmark; ^6^Department of Infectious Diseases, Odense University Hospital, Odense, Denmark; ^7^Department of Clinical Research, University of Southern Denmark, Odense, Denmark; ^8^Department of Medicine, Zealand University Hospital, Roskilde, Denmark; ^9^Department of Infectious Diseases, Copenhagen University Hospital—Amager and Hvidovre, Hvidovre, Denmark; ^10^Department of Clinical Medicine, University of Copenhagen, Copenhagen, Denmark; ^11^Deparment of Cardiology and Emergency Medicine, Herlev Hospital, Herlev, Denmark; ^12^Department of Clinical Immunology, Copenhagen University Hospital—Rigshospitalet, Copenhagen, Denmark; ^13^Department of Infectious Diseases, Copenhagen University Hospital—Rigshospitalet, Copenhagen, Denmark

**Keywords:** COVID-19, SARS-CoV-2, vaccines, antibodies, immunity, neutralization, booster, omicron

## Abstract

The SARS-CoV-2 pandemic has, as of July 2022, infected more than 550 million people and caused over 6 million deaths across the world. COVID-19 vaccines were quickly developed to protect against severe disease, hospitalization and death. In the present study, we performed a direct comparative analysis of four COVID-19 vaccines: BNT162b2 (Pfizer/BioNTech), mRNA-1273 (Moderna), ChAdOx1 (Oxford/AstraZeneca) and Ad26.COV2.S (Johnson & Johnson/Janssen), following primary and booster vaccination. We focused on the vaccine-induced antibody-mediated immune response against multiple SARS-CoV-2 variants: wildtype, B.1.1.7 (Alpha), B.1.351 (Beta), B.1.617.2 (Delta) and B.1.1.529 (Omicron). The analysis included the quantification of total IgG levels against SARS-CoV-2 Spike, as well as the quantification of antibody neutralization titers. Furthermore, the study assessed the high-throughput ACE2 competition assay as a surrogate for the traditional pseudovirus neutralization assay. The results demonstrated marked differences in antibody-mediated immune responses. The lowest Spike-specific IgG levels and antibody neutralization titers were induced by one dose of the Ad26.COV2.S vaccine, intermediate levels by two doses of the BNT162b2 vaccine, and the highest levels by two doses of the mRNA-1273 vaccine or heterologous vaccination of one dose of the ChAdOx1 vaccine and a subsequent mRNA vaccine. The study also demonstrated that accumulation of SARS-CoV-2 Spike protein mutations was accompanied by a marked decline in antibody neutralization capacity, especially for B.1.1.529. Administration of a booster dose was shown to significantly increase Spike-specific IgG levels and antibody neutralization titers, erasing the differences between the vaccine-induced antibody-mediated immune response between the four vaccines. The findings of this study highlight the importance of booster vaccines and the potential inclusion of future heterologous vaccination strategies for broad protection against current and emerging SARS-CoV-2 variants.

## Introduction

At the end of 2019, a highly transmissible, pathogenic and novel coronavirus emerged, Severe Acute Respiratory Syndrome Coronavirus 2 (SARS-CoV-2), causing the Coronavirus Disease 2019 (COVID-19) pandemic. As of July 2022, the SARS-CoV-2 pandemic has led to ∼550 million confirmed cases and caused over 6 million deaths across the world (1). Under the pressure of the COVID-19 pandemic, multiple effective vaccines were quickly developed to protect against severe disease, hospitalization and death (2–5). By July 2022, more than 12 billion COVID-19 vaccine doses had been administered globally (1).

The vaccination program against SARS-CoV-2 in Denmark started in late December 2020 with the rollout of the two mRNA-based vaccines, BNT162b2 (Pfizer/BioNTech) and mRNA-1273 (Moderna), and shortly thereafter an adenoviral vector-based vaccine, ChAdOx1 (Oxford/AstraZeneca). In March 2021, the Danish Health Authority decided to exclude ChAdOx1 from the vaccination program due to a possible link between the vaccine and a rare syndrome, now designated vaccine-induced immune thrombotic thrombocytopenia (VITT) (6, 7). Recipients of one dose of ChAdOx1 were offered heterologous vaccination with a second dose of an mRNA vaccine (BNT162b2 or mRNA-1273). Due to the risk of an equivalent link between Ad26.COV2.S (Johnson & Johnson/Janssen), another adenoviral vector-based vaccine, and VITT (7, 8), the Danish Health Authority decided to only administer Ad26.COV2.S through a voluntary system outside of the Danish national vaccination program.

The majority of COVID-19 vaccines were developed as two dose regimens (one dose for Ad26.COV2.S) and made on the basis of the original Wuhan-Hu-1 sequence of the SARS-CoV-2 Spike (S) protein (9). Since the end of 2020, a series of novel variants of concern (VOCs) have emerged, including B.1.1.7 (Alpha), B.1.351 (Beta), B.1.617.2 (Delta) and B.1.1.529 (Omicron), causing new waves of infections worldwide.

Currently, B.1.1.529 has become the dominant SARS-CoV-2 strain globally with a greater number of mutations than previous VOCs and several divergent sub-lineages (10). These mutations include 15 clustered in the receptor-binding domain region of the S protein, which is the main target of neutralizing antibodies after SARS-CoV-2 infection and vaccination. Nine of these mutations map to the angiotensin-converting enzyme 2 (ACE2) receptor-binding motif enhancing the binding affinity of ACE2 to the receptor-binding domain of B.1.1.529 (11). This leads to significantly increased transmissibility, unprecedented abilities to evade immunity by displaying almost complete resistance toward the majority of monoclonal antibodies and a substantial loss of neutralizing potency. Consequently, this reduces the efficacy of COVID-19 vaccines (12–14).

Along with documentation of waning immunity over time post-vaccination (15, 16), several studies have shown that prior SARS-CoV-2 infection and primary COVID-19 vaccination was insufficient for protection against infection with B.1.1.529. This was demonstrated by non-quantifiable neutralization titers *in vitro*, and higher rates of reinfection and vaccine breakthrough cases (13, 14, 17–19). Vaccine efficacy 3–4 months after two doses of BNT162b2 has been shown to drop from 74.4% against B.1.617.2 to 15.4% against B.1.1.529 and similar observations were shown for mRNA-1273 and ChAdOx1 (20). Administration of a booster dose was demonstrated to increase and prolong vaccine-induced neutralizing antibody potency against B.1.1.529, thus contributing to sustain control of the evolving pandemic (13, 14, 17, 18, 20).

In the present study, we performed a direct comparative analysis of vaccine-induced total immunoglobulin G (IgG) levels and antibody neutralization titers against different SARS-CoV-2 variants: wildtype (wt), B.1.1.7, B.1.351, B.1.617.2, and B.1.1.529, following primary and booster vaccination with four COVID-19 vaccines: BNT162b2, mRNA-1273, ChAdOx1 and Ad26.COV2.S, in a healthy sub-population of the Danish National Cohort Study of Effectiveness and Safety of SARS-CoV-2 vaccines (ENFORCE). Furthermore, considering the marked resistance against antibody-mediated neutralization demonstrated by B.1.1.529, the high-throughput, cell- and virus-free ACE2 competition assay was assessed as a surrogate for the pseudovirus neutralization assay. This assay has the potential of measuring lower antibody neutralization capacity with a multiplex readout of different SARS-CoV-2 variants from several samples in 1 day and without the requirement of biosafety level 2 facilities.

## Materials and methods

The Danish National Cohort Study of Effectiveness and Safety of SARS-CoV-2 vaccines (ENFORCE) was designed as an open-label, non-randomized, parallel group, phase IV study. The study enrolled adults in Denmark prior to their first COVID-19 vaccination offered through the Danish vaccination program (clinicaltrails.gov, identifier: NCT04760132). The enrollment took place at seven study sites, covering all five Danish regions, from February to August 2021. The study protocol was approved by the Danish Medicines Agency (#2020-006003-42) and the National Committee on Health Research Ethics (#1-10-72-337-20). All participants provided written informed consent. The ENFORCE cohort has previously been described by Søgaard et al. (21) and Stærke et al. (22).

The present study was a part of the ENFORCE sub-studies, with the primary objective to quantify and compare the neutralizing capacity of vaccine-induced antibodies following primary and booster doses of different COVID-19 vaccines among a healthy sub-population of the ENFORCE participants.

### Study design and data collection

This study included study participants from the ENFORCE cohort vaccinated with BNT162b2, mRNA-1273, ChAdOx1 and Ad26.COV2.S. Approximately 25 individuals from each COVID-19 vaccine group, that met the following criteria were randomly selected for inclusion in the sub-study: (1) aged from 18 to 65 years, (2) a Charlson Comorbidity Index score of zero, and (3) data collected at the third study visit (90 days ± 14 days after first vaccination). Information on age, sex, medical history, vaccine priority group, vaccination dates and vaccine type were collected and confirmed by the Danish National Patient Registry and the Danish Vaccination Registry. Serum and plasma samples drawn at the third study visit and the Xc study visit (28 days ± 8 days after booster vaccination) were used to quantify the COVID-19 vaccine-induced antibody response.

Study participants vaccinated with an mRNA vaccine, BNT162b2 or mRNA-1273, received a booster vaccine homologous to the primary vaccine, while participants vaccinated with an adenoviral vector vaccine, ChAdOx1 or Ad26.COV2.S, received an mRNA booster vaccine.

Blood samples from SARS-CoV-2 recovered individuals, infected with SARS-CoV-2 wt at the start of the pandemic (March/April 2020), were collected as part of the CoroNAT study and were used herein as convalescent comparators. The CoroNAT study protocol was approved by the National Committee on Health Research Ethics (#1-10-72-76-20). All participants provided written informed consent (23). Individuals with verified SARS-CoV-2 infection, defined as Spike IgG positive at enrollment (data from Statens Serum Institut) or any previous positive SARS-CoV-2 PCR (data extracted from the Key Infectious Diseases System database and the Danish National Microbiology database) were excluded from the vaccine comparison.

### Quantification of SARS-CoV-2-spike IgG

To detect and quantify IgG responses against multiple SARS-CoV-2 VOCs, we utilized a highly sensitive, electro chemiluminescent immunoassay from Meso Scale Discovery (MSD) (Meso Scale Diagnostics LLC, Maryland, USA). Multi-spot, 96-well, V-PLEX plates coated with purified antigens were used for the detection of IgG antibodies against SARS-CoV-2-Spike (SARS-CoV-2-S) wt (Wuhan-Hu-1), B.1.1.7, B.1.351, B.1.617.2, and B.1.1.529; BA.1, BA.2, and BA.3 [V-PLEX SARS-CoV-2 Panel 13 (IgG) kit (K15463U-2) and Panel 25 (IgG) kit (K15583U-2)]. The assays were performed according to the manufacturer’s protocol.

Serum or plasma samples were diluted 1:5,000 in diluent buffer, along with a fourfold seven-point dilution of the reference standard and a blank. Plates were read on a MESO SECTOR S600 Reader. Raw data was processed by MSD Discovery Workbench Software (Version 4.0). Total IgG concentrations were calculated by fitting the electro chemiluminescence signals to the corresponding calibration curves. Quantifications were reported in units per mL (U/mL).

### Production of SARS-CoV-2 pseudoviruses

Pseudoviruses with SARS-CoV-2-S were produced according to methods previously described by Nielsen et al. (23). Sub-confluent HEK-293T cells were transfected by polyethylenimine with the S protein expressing plasmid (pCG1-SARS-CoV-2-S wt (Wuhan-Hu-1 including D614G), B.1.1.7, B.1.351, B.1.617.2, and B.1.1.529; BA.1) for 18 h. Following, the cells were transduced with VSV-ΔG pseudovirus (vesicular stomatitis virus which lacks the VSV glycoprotein gene) expressing a green fluorescent protein (GFP) reporter gene (multiplicity of infection = 3). After 2 h of infection, the cells were washed to remove residual virus and fresh medium added. Exceptionally, for the production of pseudoviral particles incorporating the S protein of B.1.1.529; BA.1, anti-VSV-G from I1 hybridoma cells was added to the medium to neutralize remaining virus. Supernatants were collected after 24 h, centrifuged, aliquoted and stored at –80°C. A VSV-ΔG-mock was produced synchronously to allow subtraction of any background.

### Pseudovirus neutralization assay

To determine the neutralizing potency of COVID-19 vaccine-induced antibodies, we performed a neutralization assay with VSV-ΔG-SARS-CoV-2-S pseudovirus. Heat-inactivated plasma samples were fivefold eight-point diluted in medium and mixed with the pseudovirus for 1 h. Sub-confluent Vero76 c-myc cells expressing human TMPRSS2 (Transmembrane Serine Protease 2) were incubated with plasma and pseudovirus for ∼18 h, yielding a final plasma dilution of 1:25-1:1,953,125. The cells were washed, trypsinized and fixed in 2% paraformaldehyde, before GFP expression was determined on a Miltenyi Biotec MACSquant 16 flow cytometer. All samples were run in duplicates and virus-only positive controls and cell-only negative controls were included in each assay. The VSV-ΔG-mock background signal was subtracted from all samples.

The measured GFP expression was analyzed using FlowJo (Version 10.8.0). The half maximal neutralization titers (NT50) were reported as the plasma dilution at which infectivity of the pseudovirus was inhibited by 50% relative to the virus-only positive controls. NT50 values were calculated using an inhibitor vs. dose-response curve fit with non-linear regression with a hill slope of –1.0 by GraphPad Prism Software (Version 9.3.1). NT50 was non-quantifiable in cases of less than 95% inhibition of infection in the wells of the least diluted plasma, 1:25. All samples with non-quantifiable NT50 values or calculated values < 25 were adjusted to the lowest plasma dilution factor, NT50 = 25.

### Quantification of ACE2 receptor blocking

A multiplexed MSD immunoassay was used to measure the ability of vaccine-induced antibodies in serum or plasma to block ACE2 binding to SARS-CoV-2-S. Thereby evaluating the functional potential of neutralizing antibodies to compete with the ACE2 receptor for binding to SARS-CoV-2-S. Multi-spot, 96-well, V-PLEX plates coated with SARS-CoV-2-S wt (Wuhan-Hu-1), B.1.1.7, B.1.351, B.1.617.2, and B.1.1.529; BA.1, BA.2, and BA.3, were used for the quantification of ACE2 receptor blocking [V-PLEX SARS-CoV-2 Panel 13 (ACE2) kit (K15466U-2) and Panel 25 (ACE2) kit (K15586U-2)]. The assays were performed according to the manufacturer’s protocol.

Serum or plasma samples were diluted 1:10 and 1:100 in diluent buffer. For panel 13 assays, an ACE2 calibration reagent provided by the manufacturer was added, but no calibration reagent was provided for panel 25. Plates were read on a MESO SECTOR S600 Reader. Raw data was processed by MSD Discovery Workbench Software (Version 4.0). Quantifications were reported in U/mL and percentage of ACE2 receptor blocking for panel 13 and in percentage of ACE2 receptor blocking for panel 25.

### Data and statistical analysis

Demographic characteristics at enrollment of the included participants in this study were analyzed by Chi-squared tests (categorical variables) and one-way ANOVA tests (continuous variables).

Boxplots, showing the median along with the lower and upper quartiles, as well as error bars indicating 95% CI, were used to present all data. Data obtained from MSD immunoassays and pseudovirus neutralization assays were compared using Mann-Whitney tests (two groups) and Kruskal-Wallis tests (≥ three groups). Wilcoxon tests were used to compare the effect of a booster dose. All statistical tests were followed by a *post hoc* Dunn’s multiple comparisons test adjusted using Bonferroni correction. *P*-values ≤ 0.05 were considered statistically significant. *P*-values were denoted as follows: * = *p* ≤ 0.05, ^**^ = *p* < 0.01, ^***^ = *p* < 0.001, and ^****^ = *p* < 0.0001.

Spearman’s rank correlation test was used to assess the correlation between NT50 values measured by the pseudovirus neutralization assay and ACE2 receptor blocking measured by the ACE2 competition assay.

Data analysis and visualization was conducted in R (Version 4.0.4) and RStudio Desktop (Version 1.4.1106).

## Results

In a direct comparison, we assessed the capacity of four COVID-19 vaccines to produce Spike-specific (S-specific) antibodies and induce antibody-mediated neutralization of SARS-CoV-2-S. A total of 96 healthy individuals from the ENFORCE cohort were included in this sub-study. However, to focus on the vaccine-induced antibody response, we excluded previously infected individuals (*n* = 11), eliminating the impact of antibodies generated by previous infection. The vaccine-type comparative analysis included 85 individuals (61.2% females): 22 vaccinated with two doses of BNT162b2 (median age of 54 years), 24 vaccinated with two doses of mRNA-1273 (median age of 54.5 years), 20 heterologous vaccinated with one dose of ChAdOx1 and a second dose of an mRNA vaccine (median age of 45 years), and 19 vaccinated with one dose of Ad26.COV2.S (median age of 33 years). A total of 25 SARS-CoV-2 recovered individuals infected with the original SARS-CoV-2 variant (median age of 47 years) were used in this study as convalescent comparators. The demographic characteristics of the study participants at enrolment are shown in Table 1.

**TABLE 1:** Demographic characteristics.

	BNT162b2 (*N* = 22)	mRNA-1273 (*N* = 24)	ChAdOx1/mRNA (*N* = 20)	Ad26.COV2.S (*N* = 19)	Convalescent (*N* = 25)	Overall (*N* = 110)
**Sex**						
Male	8 (36.4%)	12 (50.0%)	5 (25.0%)	9 (47.4%)	20 (80.0%)	54 (49.1%)
Female	14 (63.6%)	12 (50.0%)	15 (75.0%)	10 (52.6%)	5 (20.0%)	56 (50.9%)
**Age (years)**						
Median [min, max]	54.0 [19.0, 64.0]	54.5 [40.0, 63.0]	45.0 [19.0, 60.0]	33.0 [23.0, 47.0]	47.1 [26.0, 67.8]	48.0 [19.0, 67.8]
**Priority group**						
Health care professionals	9 (40.9%)	0 (0%)	20 (100%)	0 (0%)	4 (16%)	33 (30%)
General population	13 (59.1%)	24 (100%)	0 (0%)	19 (100%)	21 (84%)	77 (70%)
**Days from 1st vaccine to 3rd study visit**						
Median [min, max]	92.5 [83.0, 102]	91.0 [79.0, 99.0]	98.5 [84.0, 114]	92.0 [42.0, 101]	108* [75.0, 119]	95.5 [42.0, 119]
**Days from 2nd Vaccine to 3rd study visit**						
Median [min, max]	62.0 [45.0, 79.0]	56.0 [44.0, 64.0]	19.0 [7.00, 35.0]	NA [NA, NA]	NA [NA, NA]	54.0 [7.00, 79.0]

* Days from confirmed positive SARS-CoV-2 PCR to study visit.

**Table:** 

**Booster dose evaluation**	**BNT162b2** **(*N* = 11)**	**mRNA-1273** **(*N* = 12)**	**ChAdOx1/mRNA** **(*N* = 5)**	**Ad26.COV2.S/mRNA** **(*N* = 6)**	**(*N* = 34)**
**Sex**					
Male	5 (45.5%)	5 (41.7%)	1 (20.0%)	2 (33.3%)	13 (38.2%)
Female	6 (54.5%)	7 (58.3%)	4 (80.0%)	4 (66.7%)	21 (61.8%)
**Age (years)**					
Median [min, max]	55.0 [22.0, 64.0]	54.5 [48.0, 62.0]	55.0 [31.0, 60.0]	27.0 [23.0, 34.0]	54.0 [22.0, 64.0]
**Days from 2nd vaccine to booster vaccine**					
Median [min, max]	190 [146, 273]	169 [161, 204]	163 [160, 189]	161** [128,169]	170 [128, 273]
**Days from booster vaccine to Xc study visit**					
Median [min, max]	28.0 [21.0, 75.0]	29.5 [22.0, 45.0]	26.0 [21.0, 30.0]	25.0 [15.0, 41.0]	27.5 [15.0, 75.0]

**Days from 1st vaccine to booster vaccine.

### Levels of SARS-CoV-2-S IgG after COVID-19 vaccination

Serum samples were collected at the third study visit (90 days ± 14 days after first vaccination) and SARS-CoV-2-S IgG antibodies were quantified.

**FIGURE 1:**
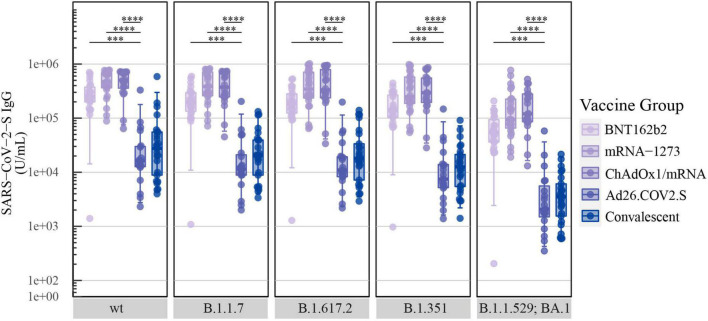
Levels of SARS-CoV-2-S IgG after COVID-19 vaccination: Levels of total SARS-CoV-2-S IgG in U/mL induced by primary COVID-19 vaccination with BNT162b2 (*n* = 22), mRNA-1273 (*n* = 24), ChAdOx1/mRNA (*n* = 20) or Ad26.COV2.S (*n* = 19) quantified by the MSD platform (serum 1:5,000). Data from convalescent comparators (*n* = 25) are also displayed, but are not included in the statistical analysis. From left to right: SARS-CoV-2-S wt (Wuhan-Hu-1) and the following SARS-CoV-2-S VOCs: B.1.1.7 (Alpha), B.1.617.2 (Delta), B.1.351 (Beta) and B.1.1.529; BA.1 (Omicron). The boxplots present the lower quartile, median and upper quartile, and the error bars indicate 95% CI. *P*-values were indicated as follows: ^***^p < 0.001 and ^****^p < 0.0001.

The median IgG antibody levels specific for SARS-CoV-2-S wt were highest for ChAdOx1/mRNA: 503,992 U/mL [IQR: 359,170-709,457] followed by mRNA-2173: 471,670 U/mL [364,131–692,740] and BNT162b2: 251,511 U/mL [199,365–376,470]. In contrast, significantly lower IgG antibody levels were detected for Ad26.COV2.S: 16,241 U/mL [12,664–29,986], which were comparable with the convalescent individuals: 27,497 U/mL [8,875–55,419] (Figure 1).

The quantification of SARS-CoV-2-S IgG specific for B.1.1.7, B.1.617.2, and B.1.351 demonstrated similar vaccine-induced antibody responses as observed for SARS-CoV-2-S wt. Generally, all vaccine recipients had slightly lower levels of B.1.1.529; BA.1 S-specific IgG compared with the other variants. As observed for SARS-CoV-2-S wt, the quantifications displayed significantly higher median antibody titers for BNT162b2, mRNA-1273 and ChAdOx1/mRNA compared with Ad26.COV2.S recipients for all included variants (Figure 1).

### Neutralizing antibody responses to pseudoviral SARS-CoV-2-S after COVID-19 vaccination

Plasma samples collected at the third study visit were used to analyze the neutralizing capacity of vaccine-induced antibodies by a pseudovirus neutralization assay employing VSV-ΔG-SARS-CoV-2-S pseudovirus and NT50 values were determined.

**FIGURE 2:**
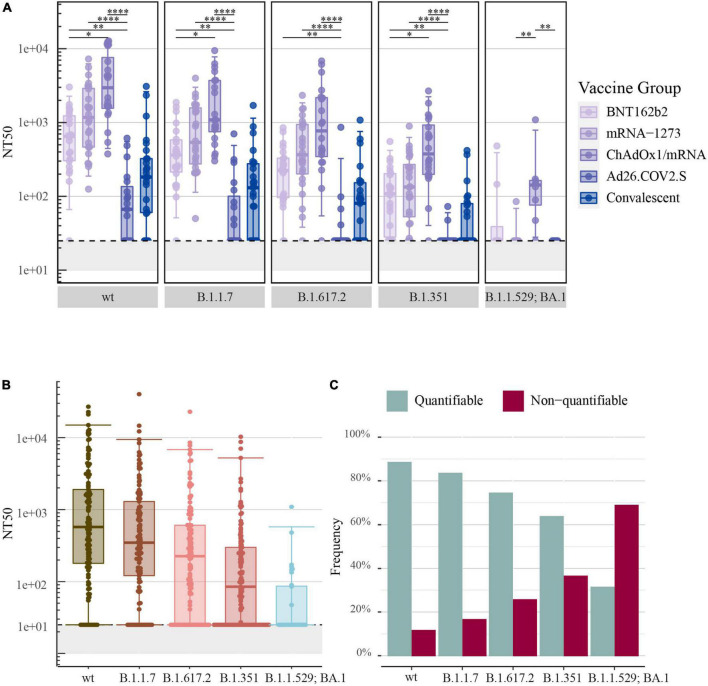
Neutralizing antibody responses to pseudoviral SARS-CoV-2-S after COVID-19 vaccination: **(A)** The 50% neutralization titers (NT50) induced by primary COVID-19 vaccination with BNT162b2 (*n* = 22), mRNA-1273 (*n* = 24), ChAdOx1/mRNA (*n* = 20) or Ad26.COV2.S (*n* = 19) quantified by the pseudovirus neutralization assay. Data from convalescent comparators (*n* = 25) are also displayed, but are not included in the statistical analysis. **(B)** NT50 values merged for all vaccine types. **(C)** The frequency of quantifiable (> 25) and non-quantifiable (≤ 25) NT50 values merged for all vaccine types. From left to right: SARS-CoV-2-S wt (Wuhan-Hu-1 including D614G) and the following SARS-CoV-2-S VOCs: B.1.1.7 (Alpha), B.1.617.2 (Delta), B.1.351 (Beta) and B.1.1.529; BA.1 (Omicron) (B.1.1.529; BA.1, *n* = 32: eight individuals per vaccine group). All boxplots present the lower quartile, median and upper quartile, and the error bars indicate 95% CI. *P*-values were indicated as follows: *p ≤ 0.05, ^**^p < 0.01, and ^****^p < 0.0001.

Correspondingly, as demonstrated for SARS-CoV-2-S IgG levels, the highest NT50 values for SARS-CoV-2-S wt were determined for recipients of ChAdOx1/mRNA: median: 4,292 [IQR: 1,639–11,377] followed by mRNA-1273: 1,285 [466–3,078] and BNT162b2: 643 [300–1,278]. Significantly lower NT50 values were determined for recipients of Ad26.COV2.S: 79 [25–182], which were on par with the convalescent comparators: 182 [60–324]. Additionally, significantly higher NT50 values were observed in the ChAdOx1/mRNA vaccine group compared with the BNT162b2 vaccine group (*P*-value = 0.012) (Figure 2A).

The assessment of SARS-CoV-2-S neutralizing antibodies specific for B.1.1.7, B.1.617.2, and B.1.351 showed a similar order of vaccine-induced neutralizing antibody responses as observed for SARS-CoV-2-S wt. The analysis of SARS-CoV-2-S B.1.1.529; BA.1 showed lower NT50 values and did not display a similar ranking of neutralizing antibody responses (Figure 2A).

All data was merged irrespectively of vaccine type and antibody neutralization capacity was assessed with focus on the different SARS-CoV-2 variants. The highest NT50 values were observed for SARS-CoV-2-S wt: 577 [180–1,906], while NT50 values decreased progressively with an increasing number of S protein mutations [B.1.1.7: 348 (121–1,296), B.1.617.2: 225 (25–610), B.1.351: 84 (25–300) and B.1.1.529; BA.1: 25 (25–86)] (Figure 2B).

The pseudovirus neutralization assay used a lowest plasma dilution factor of 1:25. The assay was therefore unable to determine antibody neutralization capacity for samples with poor neutralizing activity. In compliance with a decrease in antibody neutralization capacity, a higher frequency of non-quantifiable NT50 values was observed with an increasing number of S protein mutations. Consequently, 69% of samples analyzed for B.1.1.529; BA.1 were below the assay cut-off of 25-fold dilution (Figure 2C).

### ACE2 competition assay as a surrogate for quantifying antibody neutralization capacity

The pseudovirus neutralization assay facilitated examination of the neutralizing potency of antibodies. However, the assay was unable to estimate the low antibody neutralization titers observed for B.1.1.529. In consequence, an ACE2 competition assay was assessed as a surrogate for the pseudovirus neutralization assay with the potential of measuring vaccine-induced antibody neutralization capacity at lower levels. Serum samples were therefore used to quantify the neutralizing capacity of vaccine-induced antibodies reported as SARS-CoV-2-S ACE2 receptor-blocking antibodies in U/mL and as percentage of ACE2 receptor blocking.

**FIGURE 3:**
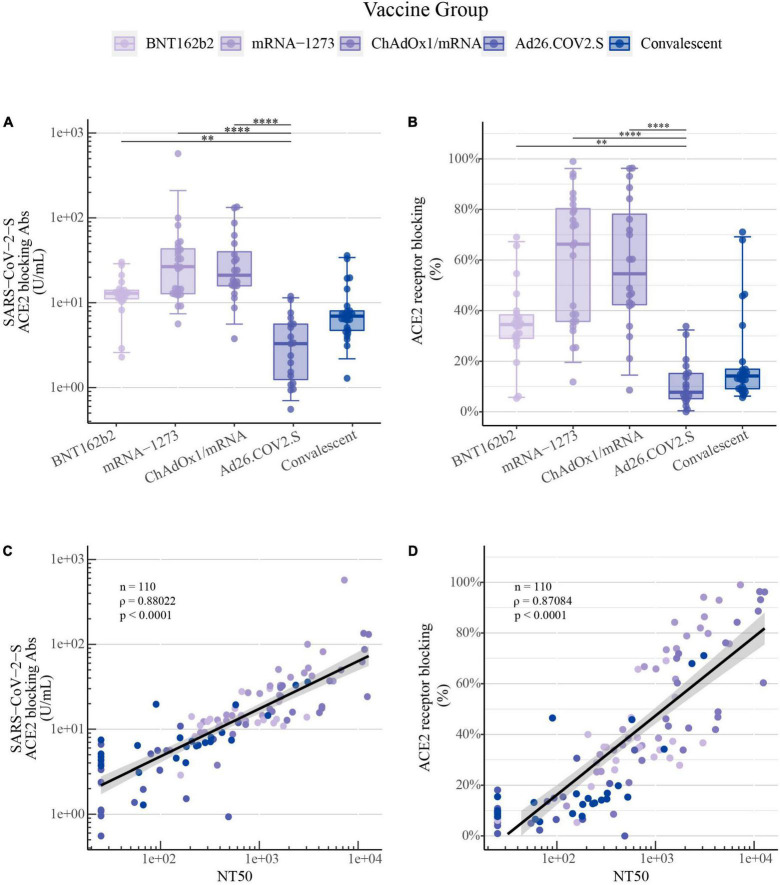
ACE2 competition assay as a surrogate for quantifying COVID-19 vaccine-induced antibody neutralization capacity: **(A)** SARS-CoV-2-S ACE2 receptor-blocking antibodies in U/mL and **(B)** ACE2 receptor blocking in percentage for SARS-CoV-2-S wt induced by primary COVID-19 vaccination with BNT162b2 (*n* = 22), mRNA-1273 (*n* = 24), ChAdOx1/mRNA (*n* = 20) or Ad26.COV2.S (*n* = 19) quantified by the MSD platform (serum 1:100). Data from convalescent comparators (*n* = 25) are also displayed, but are not included in the statistical analysis. The boxplots present the lower quartile, median and upper quartile, and the error bars indicate 95% CI. *P*-values were indicated as follows: **p < 0.01 and ****p < 0.0001. **(C)** Spearman’s correlation between SARS-CoV-2-S wt NT50 values quantified by the pseudovirus neutralization assay and ACE2 receptor-blocking antibodies in U/mL and **(D)** ACE2 receptor blocking in percentage quantified by the MSD ACE2 competition assay.

In comparison to the findings of SARS-CoV-2-S wt IgG levels and NT50 values, a similar ranking of vaccine-induced responses against SARS-CoV-2-S wt was detected utilizing the ACE2 competition assay for both ACE2 receptor-blocking antibodies in U/mL and percentage of ACE2 receptor blocking (Figures 3A,B, respectively). A very strong positive correlation was observed for SARS-CoV-2 wt between NT50 values quantified by the pseudovirus neutralization assay and the ACE2 competition assay for both the calculated concentration of ACE2 receptor-blocking antibodies (ρ = 0.88, *P*-value < 0.0001, Figure 3C) and for the percentage of ACE2 receptor blocking (ρ = 0.87, *P*-value < 0.0001, Figure 3D).

This strong positive correlation between pseudovirus neutralization and the calculated concentration of ACE2 receptor-blocking antibodies both in U/mL or the percentage of ACE2 receptor blocking was also observed for SARS-CoV-2 S-specific for B.1.1.7, B.1.617.2, and B.1.351 (Supplementary Figures 1A,B, respectively).

**FIGURE 4:**
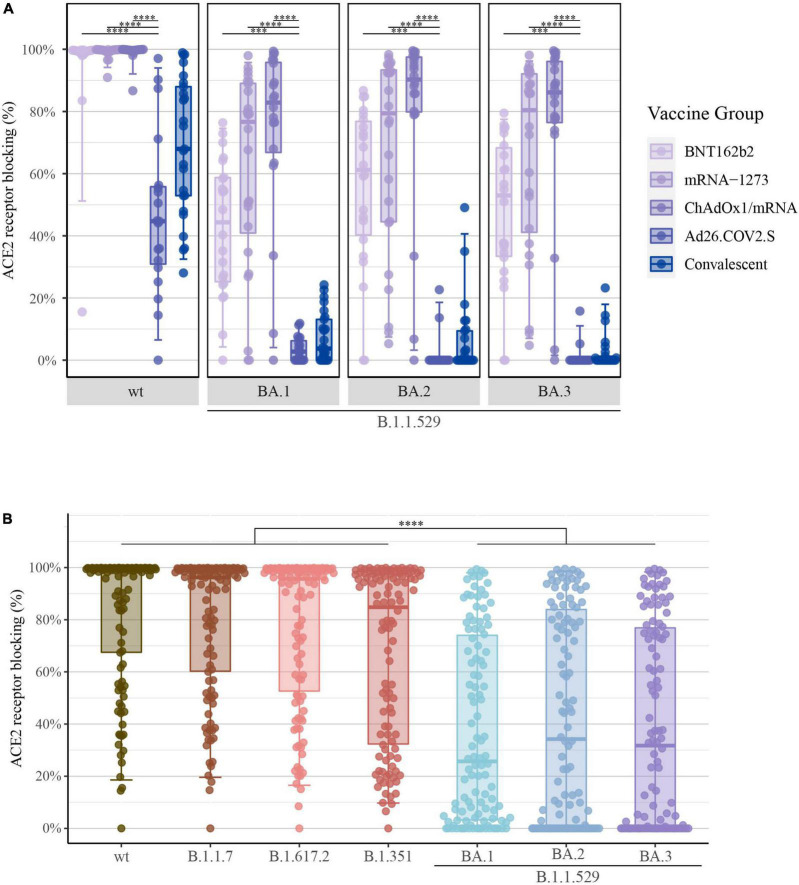
Percentage of ACE2 receptor blocking after COVID-19 vaccination: **(A)** Percentage of ACE2 receptor blocking induced by primary COVID-19 vaccination with BNT162b2 (*n* = 22), mRNA-1273 (*n* = 24), ChAdOx1/mRNA (*n* = 20) or Ad26.COV2.S (*n* = 19) quantified by the MSD platform (serum 1:10). Data from convalescent comparators (*n* = 25) are also displayed, but are not included in the statistical analysis. From left to right: SARS-CoV-2-S wt (Wuhan-Hu-1) and B.1.1.529; BA.1, BA.2 and BA.3 (Omicron). **(B)** Percentage of ACE2 receptor blocking merged for all vaccine types. From left to right: SARS-CoV-2-S wt, B.1.1.7 (Alpha), B.1.617.2 (Delta), B.1.351 (Beta), and B.1.1.529; BA.1, BA.2 and BA.3 (Omicron). All boxplots present the lower quartile, median and upper quartile, and the error bars indicate 95% CI. *P*-values were indicated as follows: ***p < 0.001 and ****p < 0.0001.

The ACE2 competition assay was utilized to measure antibody neutralization capacity for SARS-CoV-2-S B.1.1.529; BA.1, BA.2, and BA.3. The quantification of ACE2 receptor blocking demonstrated the same ranking of vaccine-induced responses as shown previously. As demonstrated for the levels of SARS-CoV-2-S IgG and NT50 values, the ACE2 competition data showed significantly higher percentages of ACE2 receptor blocking in individuals vaccinated with either one of the two mRNA vaccines or with ChAdOx1/mRNA compared with Ad26.COV2.S recipients (Figure 4A).

Further, when merging all data irrespectively of vaccine type, a significant reduction in the percentage of ACE2 receptor blocking was observed for B.1.1.529 [BA.1: 25.7% (IQR: 3–74), BA.2: 34.2% (0–84) and BA.3: 31.7% (0–77)] compared with previous SARS-CoV-2 VOCs [wt: 98.9% (67–100), B.1.1.7: 96.2% (60–99), B.1.617.2: 95.7% (53–99) and B.1.351: 84.8% (32–98)] (*P*-value < 0.0001) (Figure 4B). Again, these findings demonstrate that accumulation of S protein mutations was accompanied by a gradual decline of vaccine-induced neutralizing antibody capacity.

### Levels of SARS-CoV-2-S IgG and ACE2 receptor blocking after COVID-19 booster vaccination

Serum samples were collected at the Xc study visit (28 days ± 8 days after booster vaccination) and SARS-CoV-2-S IgG levels and the percentage of ACE2 receptor blocking was quantified.

**FIGURE 5:**
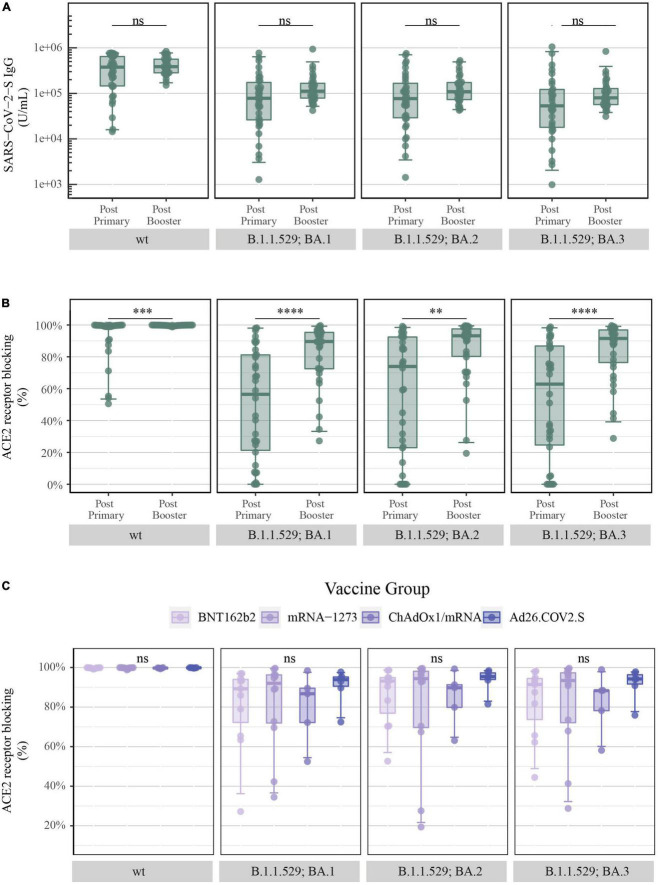
Levels of SARS-CoV-2-S IgG and percentage of ACE2 receptor blocking after COVID-19 booster vaccination: **(A)** Levels of total SARS-CoV-2-S IgG in U/mL and **(B)** ACE2 receptor blocking in percentage at the third study visit (after primary vaccination) and at the Xc study visit (after booster vaccination) merged for all vaccine types quantified by the MSD platform (after primary = serum and after booster = plasma, IgG = 1:5,000 and ACE2 = 1:10). From left to right: SARS-CoV-2-S wt (Wuhan-Hu-1) and B.1.1.529; BA.1, BA.2 and BA.3 (Omicron). **(C)** ACE2 receptor blocking in percentage after booster vaccination with BNT162b2 (*n* = 11), mRNA-1273 (*n* = 12), ChAdOx1/mRNA (*n* = 5) and Ad26.COV2.S/mRNA (*n* = 6) quantified by the MSD platform (plasma 1:10). From left to right: SARS-CoV-2-S wt and B.1.1.529; BA.1, BA.2, and BA.3. All boxplots present the lower quartile, median and upper quartile, and the error bars indicate 95% CI. *P*-values were indicated as follows: ns = *p* > 0.05, ^**^p < 0.01, ^***^p < 0.001, and ^****^p < 0.0001.

The booster vaccination caused a small increment in SARS-CoV-2-S IgG levels of B.1.1.529 sub-variants (B.1.1.529; BA.1: primary vaccination: 78,014 vs. booster vaccination: 111,694 U/mL, BA.2: 77,074 vs. 109,595 U/mL and BA.3: 53,315 vs. 79,917 U/mL) (Figure 5A). In concordance with the increase of S-specific IgG following administration of a booster vaccine, a significant increase was observed in ACE2 receptor blocking of B.1.1.529 sub-variants (B.1.1.529; BA.1: 56.5 vs. 89.6%, BA.2: 73.9 vs. 93.2% and BA.3: 62.8 vs. 91.6%) (Figure 5B).

Additionally, when assessing the vaccine-induced antibody-mediated immune response after administration of a booster dose, all previously displayed vaccine-induced differences were no longer present. SARS-CoV-2-S IgG levels and antibody neutralization titers in the form of percentage of ACE2 receptor blocking were equalized, in such manner that no significant differences were observed between the four COVID-19 vaccine-induced antibody responses for both SARS-CoV-2-S wt and B.1.1.529 sub-variants (Supplementary Figure 2 and Figure 5C, respectively).

## Discussion

In this study, we presented a direct comparative analysis of four COVID-19 vaccines: BNT162b2, mRNA-1273, ChAdOx1 and Ad.26COV2.S, following primary and booster vaccination, focusing on the vaccine-induced antibody-mediated immune response against diverse SARS-CoV-2 variants: wt, B.1.1.7, B.1.617.2, B.1.351, and B.1.1.529; BA.1, BA.2, and BA.3.

This study demonstrated significantly higher SARS-CoV-2-S IgG levels and antibody neutralization titers in individuals vaccinated with BNT162b2, mRNA-1273 and ChAdOx1/mRNA compared with recipients of Ad26.COV2.S for all SARS-CoV-2 variants. We also showed that accumulation of S protein mutations of SARS-CoV-2 was accompanied by a gradual decline in antibody neutralization capacity, particularly demonstrating a marked decline against B.1.1.529. In addition, administration of a booster vaccine was shown to induce increasing levels of SARS-CoV-2-S IgG and a higher percentage of ACE2 receptor blocking against B.1.1.529 sub-variants. The vaccine-type comparative analysis after administration of a booster dose showed that the vaccine-induced SARS-CoV-2-S IgG levels and antibody neutralization titers reached similar levels, to the point were no significant differences between the four COVID-19 vaccines were detected.

All four COVID-19 vaccines evaluated in this study have been administered to reduce the incidence of COVID-19 infections and have been invaluable in reducing and preventing severe disease, hospitalization and death. Phase three trials have demonstrated that all four vaccines have high clinical efficacy against the original SARS-CoV-2 variant with mRNA-based vaccines demonstrating greater efficacy than adenoviral vector-based vaccines (3–5, 24).

The vaccine efficacy of BNT162b2 and mRNA-1273 was nearly equivalent in phase three trials, though subsequent real-world vaccine studies, including our study, have shown higher S-specific IgG levels and more pronounced antibody neutralization potency after two doses of mRNA-1273 compared with BNT162b2 (21, 25–27). This difference may be explained by several factors, including variation in the composition of the lipid nanoparticles for packaging and delivery, the mRNA dose content (30 μg for BNT162b2 and 100 μg for mRNA-1273) and/or the recommended time interval between the two primary vaccine doses (21 days for BNT162b2 and 28 days for mRNA-1273) (3, 24, 28).

Adenoviral vector-based vaccines have demonstrated lower vaccine efficacy compared with mRNA-based vaccines. However, in this study, heterologous vaccination with one dose of ChAdOx1 and a second dose of an mRNA vaccine was shown to induce high levels of SARS-CoV-2-S IgG and high antibody neutralizing titers. This observation can support other studies, including a Swedish study, that showed heterologous ChAdOx1/mRNA vaccine efficacy against symptomatic infection of 68%, which was significantly greater than the 50% efficacy of homologous ChAdOx1 vaccination (29). Furthermore, additional studies have reported superior immune responses with higher levels of S-specific IgG, neutralizing antibodies and T cell reactivity, inducing a significantly broader and highly potent immune response following heterologous relative to homologous vaccination (30–32). Some of these studies reported that the subsequent mRNA vaccine efficiently stimulated SARS-CoV-2-specific B-cell memory that had been generated by the first dose of ChAdOx1 (33, 34).

The weakest vaccine-induced antibody-mediated immunity discovered in this study was observed in individuals vaccinated with a single dose of Ad26.COV2.S. Several other studies have demonstrated considerably lower antibody levels and neutralizing antibody titers in individuals vaccinated with Ad26.COV2.S (27, 35–37). A priming dose of Ad26.COV2.S followed by an mRNA-based booster vaccination has been demonstrated, including this study, to boost S-specific IgG levels, antibody neutralizing capacity, T cell reactivity and improve vaccine efficacy compared with homologous vaccination with Ad26.COV2.S (38, 39). Heterologous COVID-19 vaccination might provide a favorable alternative for better protection against current and emerging SARS-CoV-2 VOCs by inducing a broader and more robust antibody-mediated and cell-mediated immune profile.

The emergence of novel SARS-CoV-2 variants has repeatedly received global attention. Especially, the current VOC, B.1.1.529, has been proven to have a substantial ability to avoid vaccine-induced and convalescent immune responses, thus affecting COVID-19 protection. Levels of S-specific IgG and antibody neutralization titers have shown to correlate and be highly predictive of clinical protection against symptomatic COVID-19 (40–43). However, the minimum required titers of neutralizing antibodies to provide protection against B.1.1.529 are yet to be determined.

The importance of COVID-19 vaccines was confirmed in a study examining the antibody-mediated immune response following B.1.1.529 infection. Data demonstrated that B.1.1.529 infections in unvaccinated individuals induced a limited immune response that lacked broader effective cross-neutralizing antibodies and displayed limited neutralization of non-B.1.1.529 variants. However, B.1.1.529 breakthrough infections were demonstrated to induce high neutralization titers against all SARS-CoV-2 VOCs. Thus, B.1.1.529 infections are capable of boosting pre-existing immunity induced by vaccination that is effective against B.1.1.529 and other SARS-CoV-2 variants (44).

SARS-CoV-2 B.1.1.529 has been shown to be highly resistant to neutralizing antibodies induced by vaccination and previous infections (11–14). Consequently, an additional dose of the COVID-19 vaccine was offered to boost the immune response and sustain protection against SARS-CoV-2. Our data displayed increased levels of SARS-CoV-2-S IgG and higher antibody neutralization capacity following a booster dose, which is comparable to other studies (17, 18, 45–47). Data on vaccine efficacy likewise demonstrated that a booster vaccination provided increased protection against symptomatic infection with B.1.1.529 (20). Thus, administration of a booster dose provides great potential for improving neutralizing antibody capacity against B.1.1.529 and possible future SARS-CoV-2 VOCs.

Due to the fact that many individuals had non-quantifiable antibody neutralization titers for SARS-CoV-2 B.1.1.529 by the pseudovirus neutralization assay, an additional assay was assessed to measure the potency of B.1.1.529 S-specific neutralizing antibodies with detection sensitivity at lower levels. The pseudovirus neutralization assay is a strong tool to study functional antibody responses against a virus. However, this assay is labor intensive, requires access to biosafety level 2 facilities and the use of living cells, making the assay more difficult to standardize. In addition, the assay has a detection limit at NT50 values of 25, prohibiting the quantification of low neutralizing antibody titers. The most concentrated plasma dilution examined in the pseudovirus assay is 1:25, as cell death has been shown to confound the readout at higher plasma concentrations. The ACE2 competition assay can serve as a high-throughput alternative to the traditional pseudovirus neutralization assay. The ACE2 competition assay is provided as a 96-well microtiter plate with multi-spot panels facilitating the quantification of up to 10 different SARS-CoV-2 variants from a single, small-volume of sample. However, it should be noted that the ACE2 competition assay has a narrow dynamic range and performing a dilution series is favored to ensure that all data points fall in the quantifiable range. As demonstrated in this study, and shown by Nielsen et al. (23), a very strong positive correlation was found between the readouts of the two assays, which was true for all variants tested. Thus, the data support the ACE2 competition assay as a reliable, powerful and large-scale screening tool to measure antibody neutralization titers.

There are some limitations to consider in our study. The ChAdOx1/mRNA group mainly consisted of female healthcare workers and the timing of their second vaccination was significantly closer to the third study visit compared with the BNT162b2 and mRNA-1273 vaccine groups. As immune responses tend to peak shortly after vaccination and wane over time, this is a relevant factor when considering the higher neutralizing antibody responses detected in the ChAdOx1/mRNA group. The age distribution in the four vaccine groups is also not identical. In particular, the Ad26.COV2.S group is considerably younger as a consequence of the restrictive use of Ad26.COV2.S in Denmark. However, increasing age has been shown to correlate with lower IgG levels and antibody neutralization titers (21). Consequently, the differences in age distribution did not appear to have an impact on the vaccine-induced immune responses detected in this study. Another limitation is the relatively small and varying number of participants in each vaccine group included in the comparison of vaccine-induced antibody neutralization following booster vaccination.

This study also had some limitations in regards to the assays that were performed. We measured total levels of SARS-CoV-2-S IgG by utilizing a serum dilution of 1:5,000 as suggested by the manufacturer. However, after administration of the COVID-19 booster dose, the serum samples appeared to be insufficiently diluted and reached the upper limits of the assay. Due to this, we may only detect small increments in S-specific IgG levels after administration of the booster vaccine.

## Conclusion

In conclusion, the direct comparative analysis of vaccine-induced antibody-mediated immune responses, to a range of SARS-CoV-2 variants, demonstrated marked differences in the antibody-mediated immune responses generated by each COVID-19 vaccine. Comparing vaccine types, the study showed lower levels of total S-specific IgG and antibody neutralization titers induced by one dose of the Ad26.COV2.S vaccine, intermediate levels by two doses of the BNT162b2 vaccine, and the highest levels by two doses of the mRNA-1273 vaccine or heterologous vaccination of one dose of the ChAdOx1 vaccine and a subsequent mRNA vaccine. The accumulation of SARS-CoV-2 S protein mutations was accompanied by a marked decline in antibody neutralization capacity, especially against the current VOC, B.1.1.529. However, administration of a booster dose elevated antibody responses significantly for all vaccinated individuals against B.1.1.529. The previously detected differences in antibody-mediated immunity, between the four COVID-19 vaccines after primary vaccination, were no longer detected post-booster vaccination. These findings highlight the importance of the roll-out of booster vaccines and the potential inclusion of future heterologous vaccination strategies for broad protection against current and emerging SARS-CoV-2 VOCs to remain in control of the pandemic.

## Data availability statement

All data may be made available to researchers upon request. All data will be provided as de-identified data to comply with GDPR regulations. Requests to access the datasets should be directed to MT, marttols@rm.dk.

## Ethics statement

The studies involving human participants were reviewed and approved by the Danish National Committee on Health Research Ethics, Copenhagen, Denmark (#1-10-72-337-20) and The Danish Medicines Agency, Copenhagen, Denmark (#2020-006003-42). The patients/participants provided their written informed consent to participate in this study.

## Author contributions

JL, LØ, OS, NS, JR, DR, and MT conceptualized the work. HN, IJ, LW, TB, NS, KI, AM, MJ, KP, SO, SL, and LR performed the clinical visits and collected the samples. AH, EB, MS, SA, SRA, LD, and AJ performed the laboratory analyses. AH and EB performed the data analysis and visualization. MT supervised and led the study. AH, EB, and MT drafted the manuscript. All authors reviewed and approved the final version of the manuscript.
