# Supplementary material for: Comparison of vaccine-induced antibody neutralization against SARS-CoV-2 variants of concern following primary and booster doses of COVID-19 vaccines
Source: Front Med (Lausanne). 2022 Oct 3;9:994160. doi: 10.3389/fmed.2022.994160 (PMC9574042; doi:10.3389/fmed.2022.994160)
Supplement: Supplementary file 1 [file Data_Sheet_1.pdf]

frontiers

Supplementary Material

1 Supplementary Data

1.1 ENFORCE Study Group Members

A list of ENFORCE Study Group Members is available on the next page.

ENFORCE Study Group

Sponsor/Data and Statistical Centre: CHIP, Rigshospitalet under the direction of Professor J Lundgren.

Principal Investigator: Professor LJ Østergaard, Department of Infectious Diseases, Aarhus University and Aarhus University Hospital.

Sites (alphabetical and regional coordinators in parenthesis)

Region Hovedstaden: (T Benfield), L Krohn-Dehli, DK Petersen, Hvidovre Hospital, Hvidovre.

K Fogh, E Højmark, (K Iversen), Herlev Hospital, Herlev.

Region Midtjylland: V Klastrup, F Larsen, (NB Stærke), S Schieber, A Søndergaard, M Tousgaard, Y Yehdego, Aarhus University Hospital, Aarhus.

Region Nordjylland: J Bodilsen, (H Nielsen), KT Petersen, M Ruwald, RK Thisted, Aalborg University Hospital, Aalborg.

Region Sjælland: SF Caspersen, M Iversen, LS Knudsen, JL Meyerhoff, LG Sander, (L Wiese), Zealand University Hospital Roskilde, Roskilde.

Region Syddanmark: C Abildgaard, IK Holden, (IS Johansen), L Larsen, SO Lindvig, LW Madsen, A Øvrehus, Odense University Hospital, Odense.

Scientific Steering Committee: NA Kruse, H Lomholdt, Lægemiddelstyrelsen, TG Krause, P Valentiner-Branth, Statens Serum Institut, B Søborg, Sundhedsstyrelsen, TK Fischer, Copenhagen University, C Erikstrup, Aarhus University, SR Ostrowski, Rigshospitalet, H Nielsen, Aalborg University Hospital, IS Johansen, Odense University Hospital, LJ Østergaard (chair), M Tolstrup, NB Stærke, OS Søgaard Aarhus University Hospital, L Wiese, Zealand University Hospital Roskilde, T Benfield, Hvidovre Hospital, J Lundgren, D Raben, CHIP, Rigshospitalet.

Operational Group: H Nielsen, Aalborg University Hospital, IS Johansen, Odense University Hospital, LJ Østergaard, M Tolstrup, NB Stærke, OS Søgaard, Aarhus University Hospital, L Wiese, Zealand University Hospital Roskilde, T Benfield, Hvidovre Hospital, J Lundgren (chair), D Raben, CHIP, Rigshospitalet, E Jylling, Danske Regioner, D Hougaard, Statens Serum Institut.

Coordinating Centre: Aarhus University and Aarhus University Hospital, SD Andersen, K Lykkegaard, NB Stærke, OS Søgaard, M Tolstrup, LJ Østergaard.

ENFORCE Lab: Aarhus University Hospital: SR Andreasen, E Baerends, LL Dietz, AK Hvidt, AK Juhl, R Olesen, M Tolstrup.

Data and Statistical Centre: CHIP, Rigshospitalet: KK Andersen, W Bannister, C Bjernved, FV Esmann, E Gravholdt, CM Jensen, SF Jakobsen, ML Jakobsen, TØ Jensen, D Kristensen, J Lundgren, C Matthews, N Normand, C Olsson, D Raben, J Reekie, A Traytel.

Other contributors: AM Hvass, H Støvring, Aarhus University Hospital, Statens Serum Institut.

Funding: ENFORCE has received a grant from the Danish Ministry of Health (SUM) (legal deeds 150 28/1 2021 and 263 3/6 2021).

2 Supplementary Figures

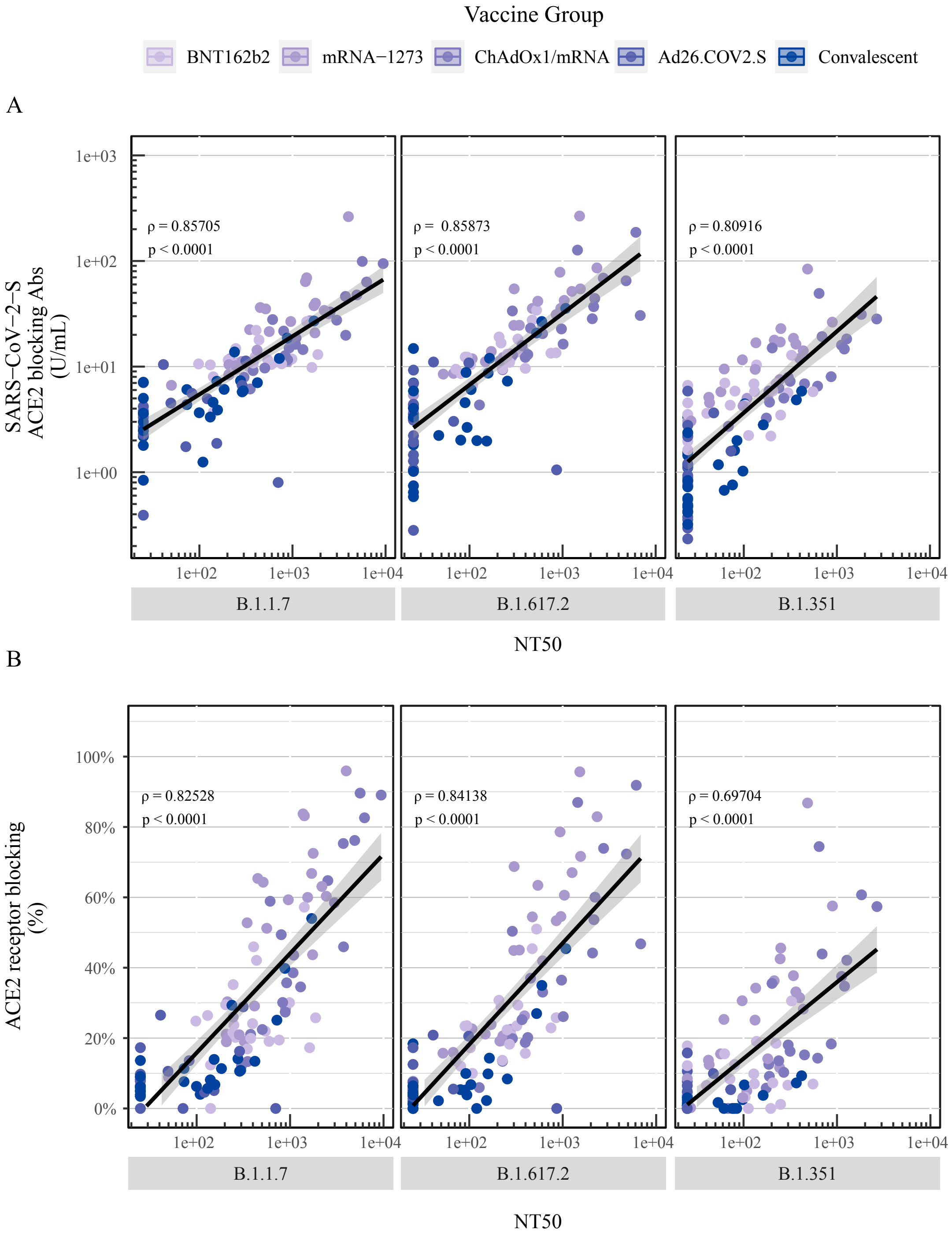

Supplementary Figure 1 - Spearman's Correlation for all SARS-CoV-2 Variants: **(A)** Spearman's correlation between SARS-CoV-2-S NT50 values quantified by the pseudovirus neutralization assay and ACE2 receptor-blocking antibodies in U/mL and **(B)** ACE2 receptor blocking in percentage quantified by the MSD ACE2 competition assay (n = 110). From left to right: SARS-CoV-2-S B.1.1.7 (Alpha), B.1.617.2 (Delta) and B.1.351 (Beta).

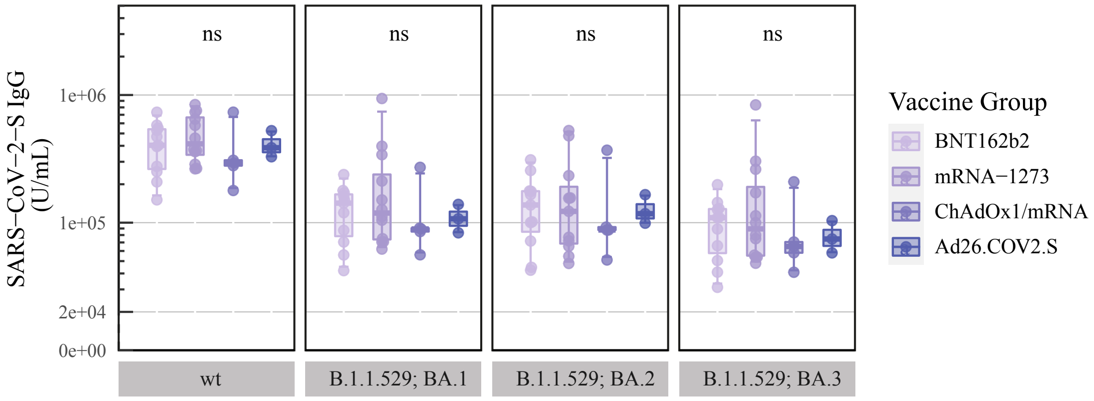

Supplementary Figure 2 – Levels of SARS-CoV-2-S IgG after COVID-19 Booster Vaccination:
Levels of total SARS-CoV-2-S IgG in U/mL after booster vaccination with BNT162b2 (n = 11), mRNA-1273 (n = 12), ChAdOx1 (n = 5) and Ad26.COV2.S (n = 6) quantified by the MSD platform (plasma 1:5,000). From left to right: SARS-CoV-2-S wt (Wuhan-Hu-1) and B.1.1.529; BA.1, BA.2 and BA.3 (Omicron). All boxplots present the lower quartile, median and upper quartile, and the error bars indicate 95% CI.
